# His Resynchronization Versus Biventricular Pacing in Patients With Heart Failure and Left Bundle Branch Block

**DOI:** 10.1016/j.jacc.2018.09.073

**Published:** 2018-12-18

**Authors:** Ahran D. Arnold, Matthew J. Shun-Shin, Daniel Keene, James P. Howard, S.M. Afzal Sohaib, Ian J. Wright, Graham D. Cole, Norman A. Qureshi, David C. Lefroy, Michael Koa-Wing, Nick W.F. Linton, Phang Boon Lim, Nicholas S. Peters, D. Wyn Davies, Amal Muthumala, Mark Tanner, Kenneth A. Ellenbogen, Prapa Kanagaratnam, Darrel P. Francis, Zachary I. Whinnett

**Affiliations:** a National Heart and Lung Institute, Imperial College London, London, United Kingdom; b St. Bartholomew’s Hospital, London, United Kingdom; c North Middlesex Hospital NHS Trust, London, United Kingdom; d Virginia Commonwealth University, Richmond, Virginia

**Keywords:** biventricular pacing, cardiac resynchronization therapy, ECGI, His bundle pacing, His resynchronization therapy, noninvasive epicardial mapping, AV, atrioventricular, BVP, biventricular pacing, CI, confidence interval, CRT, cardiac resynchronization therapy, ECG, electrocardiogram/electrocardiographic, ECGI, electrocardiographic imaging, HBP, His bundle pacing, LBBB, left bundle branch block, LV, left ventricle/ventricular, LVAT, left ventricular activation time, LVAT-95, left ventricular activation time spanning 95% of activations, LVDI, left ventricular dyssynchrony index, QRSd, QRS duration

## Abstract

**Background:**

His bundle pacing is a new method for delivering cardiac resynchronization therapy (CRT).

**Objectives:**

The authors performed a head-to-head, high-precision, acute crossover comparison between His bundle pacing and conventional biventricular CRT, measuring effects on ventricular activation and acute hemodynamic function.

**Methods:**

Patients with heart failure and left bundle branch block referred for conventional biventricular CRT were recruited. Using noninvasive epicardial electrocardiographic imaging, the authors identified patients in whom His bundle pacing shortened left ventricular activation time. In these patients, the authors compared the hemodynamic effects of His bundle pacing against biventricular pacing using a high-multiple repeated alternation protocol to minimize the effect of noise, as well as comparing effects on ventricular activation.

**Results:**

In 18 of 23 patients, left ventricular activation time was significantly shortened by His bundle pacing. Seventeen patients had a complete electromechanical dataset. In them, His bundle pacing was more effective at delivering ventricular resynchronization than biventricular pacing: greater reduction in QRS duration (−18.6 ms; 95% confidence interval [CI]: −31.6 to −5.7 ms; p = 0.007), left ventricular activation time (−26 ms; 95% CI: −41 to −21 ms; p = 0.002), and left ventricular dyssynchrony index (−11.2 ms; 95% CI: −16.8 to −5.6 ms; p < 0.001). His bundle pacing also produced a greater acute hemodynamic response (4.6 mm Hg; 95% CI: 0.2 to 9.1 mm Hg; p = 0.04). The incremental activation time reduction with His bundle pacing over biventricular pacing correlated with the incremental hemodynamic improvement with His bundle pacing over biventricular pacing (R = 0.70; p = 0.04).

**Conclusions:**

His resynchronization delivers better ventricular resynchronization, and greater improvement in hemodynamic parameters, than biventricular pacing.

Left bundle branch block (LBBB) is deleterious in patients with heart failure. Biventricular pacing improves the activation pattern, instantly shortening left ventricular activation time (LVAT) and immediately improving cardiac function. Long-term studies show substantial reduction in morbidity and mortality 1, 2.

Biventricular pacing (BVP) was first recognized as a potentially beneficial therapy from its clear hemodynamic effect (3). Relative to native LBBB, biventricular pacing (BVP) was found to shorten QRS duration (QRSd) and LVAT, earning it the moniker of “cardiac resynchronization therapy” (CRT). In fact, BVP results in the fusion of 2 nonphysiological wave fronts, resulting in only modest reductions in QRSd 4, 5, 6, 7. Computer modeling suggests that there is a large potential to deliver greater improvements in cardiac function if more effective ventricular resynchronization could be achieved (5).

His bundle pacing (HBP) has the potential to offer greater ventricular resynchronization because large reductions in QRSd have been observed when stimulating the His-Purkinje system in patients with LBBB 8, 9. How this compares with BVP is beginning to be explored 9, 10, 11 but, crucially, there is no within-patient comparison between HBP and BVP, of hemodynamic measurements alongside detailed electrical activation mapping.

In this prospective study, we tested the ability of HBP to deliver resynchronization. We then compared the electromechanical effects of His resynchronization against conventional BVP, using high-precision hemodynamic assessment (12) and noninvasive epicardial ventricular activation mapping (7).

## Methods

### His resynchronization

We defined His resynchronization as HBP that significantly shortens LVAT. We measured LVAT using noninvasive epicardial electrocardiographic imaging (ECGI). Because the margin of error of this measurement is up to 10 ms, we defined shortening as 10 ms to assess true resynchronization effects rather than measurement variation.

### Study population

Patients at a single tertiary cardiac center (Hammersmith Hospital, London, United Kingdom) scheduled for conventional biventricular pacemaker implantation with or without defibrillator were recruited. Indications for BVP were based on standard clinical criteria (13); inclusion criteria for the study were LBBB with QRSd >130 ms, ejection fraction <35%, and New York Heart Association functional class II to IV. Patients who were unable to give consent, or were clinically unstable, were excluded. All participants gave written, informed consent, and the study was approved by the local ethics committee (13/LO/1440).

### Noninvasive epicardial electrical mapping (ECGI)

Patients were fitted with a 252-electrode ECGI vest (Medtronic, Minneapolis, Minnesota) (14) before the procedure and underwent low-dose thoracic computed tomography to acquire electrode and cardiac positions. Continuous ECGI recordings were obtained throughout the hemodynamic study. From the individual electrogram activations, the left ventricular (LV) total activation time was calculated, as described in the Online Appendix. By calculating the shortest interval that spans 95% of activations recorded from the left ventricle (LVAT-95), we minimized the potential skewing of LV total activation time by noise and misannotation of outliers, while still measuring activation of almost the entire LV surface. Left ventricular dyssynchrony index (LVDI) was also calculated as the standard deviation of individual activations recorded from the LV, which has been proposed as a measure of intraventricular dyssynchrony (15). For each ventricular activation parameter, the average of 5 beats each was taken for AAI pacing, HBP, and BVP.

### Pacing

Temporary HBP was achieved via either the femoral or subclavian approach. If the femoral route was used, a quadripolar electrophysiology catheter was placed on the bundle of His, and a second catheter was positioned in the right atrial appendage for sequential atrial followed by His (AH) pacing. If the subclavian route was used, a SelectSecure 3830 lead was delivered via either a C304-His deflectable sheath or C315 fixed curve sheath (leads and delivery system: Medtronic). The lead was not actively fixated. The atrial lead for the CRT device was used to allow AH sequential pacing. The atrial and His leads were connected to the Micropace pacing system (Micropace EP, Santa Ana, California) that allowed pacing at selected AH delays. To minimize the research protocol duration during implant procedures, we did not routinely measure capture threshold or LBBB correction thresholds, because we did not actively fixate the His lead. The Micropace pacing output was programmed to 25 mA for all patients. Although this is a high output, this does not reflect the threshold of correction and furthermore Ajijola et al. (10) have demonstrated very low and stable thresholds of LBBB correction when performing permanent actively fixated His resynchronization (1.9 ± 1.2 V at 0.6 ± 0.2 ms).

HBP was attempted from the right atrium in its typical location at the anteroseptal atrioventricular (AV) groove. The His bundle was located using the electrogram (EGM) signal obtained from the catheter/lead. His bundle capture was confirmed by analysis of the 12-lead surface ECG and the catheter/lead EGM. Selective His bundle capture was differentiated from nonselective capture using standard definitions 16, 17. If pacing produced a QRS morphology similar to the intrinsic (unpaced) QRS, without shortening of the QRSd, the lead was repositioned. Data were only included for analysis if HBP resulted in a shortening of LVAT by at least 10 ms. We used this measure because it can be applied without bias (as opposed to inspection of morphology), assesses LV synchrony, and is unlikely to be affected by the presence or absence of local myocardial capture with nonselective or selective capture, respectively.

Biventricular pacing was performed via the CRT device, which was implanted using the standard clinical technique (described in full in the Online Appendix).

### Acute hemodynamic study

Invasive beat-by-beat blood pressure was recorded from the femoral or radial artery, depending on patient preference. The blood pressure signal was acquired using a transducer and amplifier (Dynascope DS-7100, Fukuda Denshi, Tokyo, Japan). Some patients agreed to the study protocol but declined the additional invasive monitoring. In these patients, noninvasive beat-by-beat blood pressure was acquired (Finapres NOVA, Finapres Medical Systems, Enschede, the Netherlands), which has been previously validated for the assessment of acute hemodynamic changes in this setting (18). Changes in this measurement correlate well with changes in invasively measured LV dP/dt (18), a measure of cardiac contractility that is largely independent of the degree of loading force. The hemodynamic data were acquired alongside surface ECG data using a data acquisition system (National Instruments, Austin, Texas) and recorded using customized software. The 12-lead ECG recordings were made using the BARD electrophysiology lab system (Boston Scientific, Natick, Massachusetts) alongside ECGI.

### High-precision hemodynamic protocol

We have previously shown the importance of performing multiple repeated hemodynamic measurements between a reference and tested setting, in order to prevent spontaneous fluctuations being misconstrued as a response to pacing therapy (19). We summarize the aspects of the protocol that generate precise estimates of hemodynamic response here and a full description is found in the Online Appendix and in our previous work employing and validating this method 12, 20, 21. We performed multiple transitions between atrial pacing (AAI), as a reference setting, and the tested pacing intervention (HBP or BVP) at a given AV delay. We compared the mean systolic blood pressure (SBP) of multiple beats immediately preceding a transition, with the mean SBP immediately after transition and calculated the difference as a response to the pacing intervention. Therefore, the mean of multiple responses (from multiple transitions) is recorded for each AV delay. Because the hemodynamic response to pacing is highly dependent on the AV delay (due to changes in LV filling), this process was repeated for multiple AV delays. This method results in at least 768 individual SBP measurements per patient being utilized in a statistically efficient way to overcome the signal-to-noise limitation of typical hemodynamic assessments with few or single hemodynamic measurements.

### Analysis of hemodynamic data

**Figure 1:**
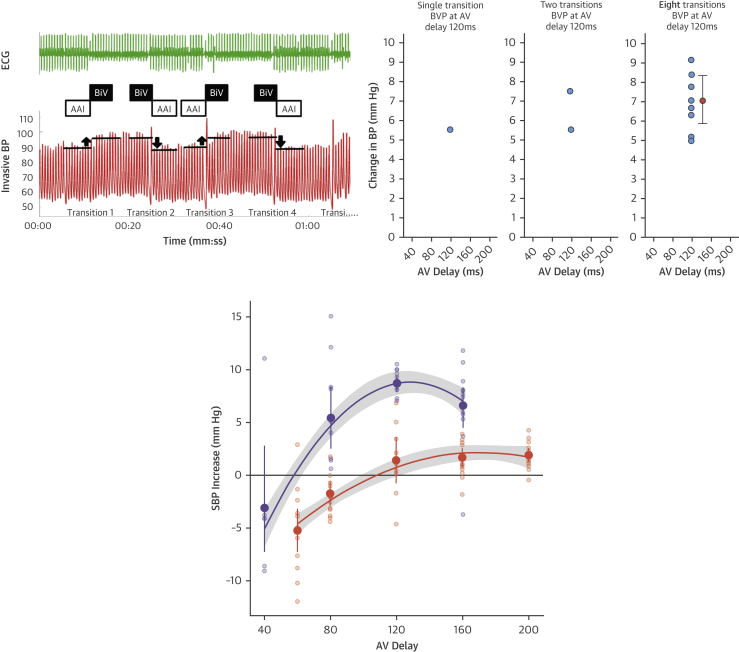
Analysis of Hemodynamic Data **(Top left)** A minimum of 4 alternations between AAI pacing and HBP or BVP were performed for each tested AV delay (therefore, a total of 8 transitions). For each alternation, relative change in SBP was calculated and the mean of the 8 transitions was calculated. **(Top right)** As more transitions occur, more data points are collected for change in SBP, eventually resulting in 10 values with mean and confidence intervals. Single or few measurements result in highly inaccurate estimates due to variability. **(Bottom)** A mean and confidence interval are calculated for each AV delay for both HBP **(purple)** and BVP **(orange)**. An example is shown of quadratic curves fitted to data from multiple transitions at a range of AV delays to produce the peak systolic blood pressure responses. AV = atrioventricular; BP = blood pressure; BVP = biventricular pacing; ECG = electrocardiogram; HBP = His bundle pacing; SBP = systolic blood pressure.

The peak blood pressure response and its CIs were calculated by fitting a quadratic curve to the data from each set of tested AV delays (Figure 1). Analysis of hemodynamic data was automated using Python, version 3.6 (Python Software Foundation, Wilmington, Delaware) so that few user inputs were required. Robust regression methods were used so that outliers did not need to be manually removed (iterated reweighted least squares and the Huber estimator using the rlm package [22]).

### Statistical analysis

In the primary analysis, we included all patients with a complete dataset, in whom the His-paced LVAT-95 was at least 10 ms shorter than during intrinsic conduction. This was selected on the basis of the standard deviation of the difference for change in LVAT-95 being <10 ms so that changes larger than this could be attributed to true resynchronization effects rather than measurement variation. Furthermore, this stringent cutoff could be applied without the potential for unintentional bias introduced by the researcher (unlike morphological analysis) and assesses LV activation regardless of whether selective or nonselective capture occurs. The power calculation for this sample size is in the Online Appendix. Baseline characteristics were tabulated and summarized with appropriate statistics. Within-patient comparisons between the hemodynamic and electrical parameters were performed using paired Student's *t*-tests. The relationship between the hemodynamic response and change in activation time was analyzed using Pearson’s correlation. Statistical analyses were performed using the statistical environment R (23) with the ggplot2 visualization package.

## Results

Twenty-three patients were recruited. In 4 patients, temporary HBP did not shorten LVAT-95 by ≥10 ms, and these patients were therefore excluded from the main analysis. In 1 patient, a technical fault prevented acquisition of ECGI data, and they were therefore also excluded from the analysis.

Eighteen patients therefore demonstrated the 10-ms LVAT-95 shortening required by our definition of His resynchronization. In 1 of them, ECGI data could be collected during His pacing, but not during BVP.

Therefore, the full dataset of ECGI and hemodynamic data for both HBP and BVP was available in 17 patients (Table 1). Six had a history of a previous myocardial infarction, and the remainder were diagnosed with nonischemic cardiomyopathy.

**Table 1:** Baseline Characteristics

Age, yrs	67 ± 10 (48–89)
Male	9 (53)
Ejection fraction, %	26 ± 7 (14–40)
NYHA functional class	2.2 ± 0.7 (1–4)
I	1 (6)
II	12 (71)
III	3 (18)
IV	1 (6)
Previous MI	6 (38)
ACE inhibitor/ARB	17 (100)
Beta-blocker	14 (82)
MRA	11 (65)
Sacubitril	2 (12)
QRS duration, ms	
Atrial pacing (AAI)	178 ± 30 (136–272)
His bundle pacing	139 ± 29 (106–200)
Biventricular pacing	158 ± 21 (109–195)
PR interval, ms	180 ± 24 (130–244)
Selective His bundle capture	2 (12)
Subclavian access His bundle pacing	13 (76)
LV lead in lateral branch of CS	17 (100)
Quadripolar LV lead	16 (94)
Invasive BP measurement	11 (65)

Values are mean ± SD (range) or n (%). The data are from 17 subjects in whom LVAT-95 was reduced by at least 10 ms and for whom data are available for both hemodynamic and electrical responses to His bundle and biventricular pacing.

ACE = angiotensin-converting enzyme inhibit; ARB = angiotensin receptor blocker; BP = blood pressure; CS = coronary sinus; LV = left ventricular; MI = myocardial infarction; MRA = mineralocorticoid receptor antagonist; NYHA = New York Heart Association.

### Electrophysiological responses

**Figure 2:**
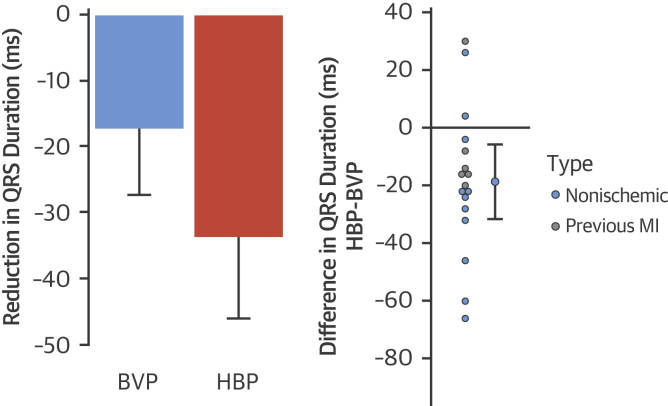
12-Lead Surface ECG QRS Responses Change in QRS duration with BVP and HBP **(left)** and within-patient incremental QRS duration reduction with HBP over BVP **(right)**. The 95% confidence intervals are displayed. MI = myocardial infarction; other abbreviations as in Figure 1.

Both HBP and BVP significantly shortened 12-lead ECG QRSd (−33.7 ms; 95% confidence interval [CI]: −46.1 to − 21.3 ms; p < 0.001; −17 ms; 95% CI: −27.3 to −7.1 ms; p = 0.002, respectively). HBP resulted in significantly more QRS shortening compared with BVP: the mean within-patient change in QRSd from BVP to HBP was −18.6 ms (95% CI: −31.6 to −5.7 ms; p = 0.007) (Figure 2).

**Figure 3:**
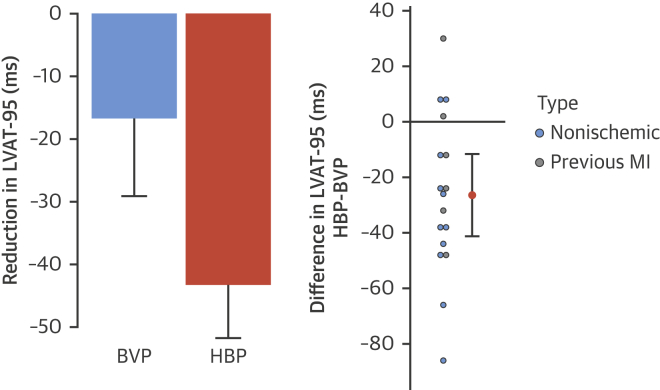
LVAT-95 Responses Change in LVAT-95 with BVP and HBP **(left)** and within-patient incremental reduction of LVAT-95 with HBP over BVP **(right)**. The 95% confidence intervals are displayed. LVAT-95 = left ventricular activation time spanning 95% of activations; other abbreviations as in Figures 1 and 2.

Using noninvasive epicardial mapping, both His bundle and BVP significantly reduced LVAT-95 compared with intrinsic activation (−43.3 ms; 95% CI: −51.7 to −34.8 ms; p < 0.001; −16.7 ms; 95% CI: −29.1 to −4.4 ms; p = 0.01, respectively). HBP reduced LVAT-95 significantly more than BVP: the mean within-patient change in LVAT-95 from BVP to HBP was −26.4 ms (95% CI: −41.2 to −11.6 ms; p = 0.02) (Figure 3). Using a 1-way analysis of variance, the standard deviation of LVAT-95 measurements during HBP was 6.6 ms, and the standard deviation of the difference for change in LVAT-95 was 9.2 ms.

**Figure 4:**
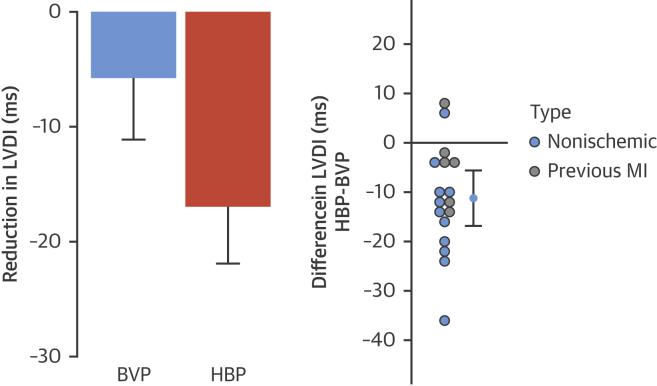
LVDI Responses Change in LVDI with BVP and HBP **(left)** and within-patient incremental reduction of LVDI with HBP over BVP **(right)**. The 95% confidence intervals are displayed. LVDI = left ventricular dyssynchrony index; other abbreviations as in Figures 1 and 2.

**Figure 5:**
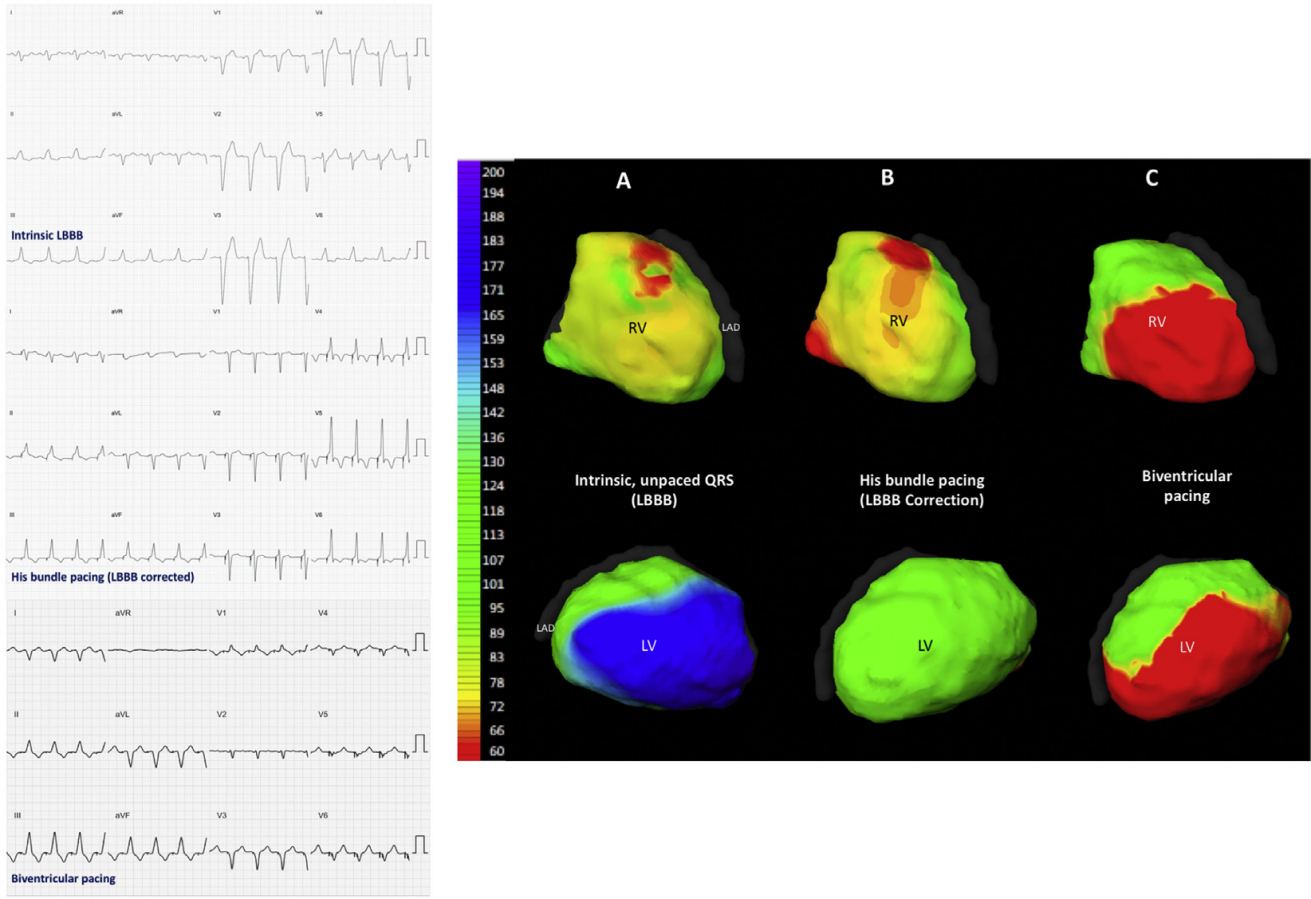
12-Lead Surface ECGs and ECGI Activation Maps **(Left)** 12-lead surface ECGs of intrinsic LBBB, His bundle pacing correction of LBBB, and biventricular pacing in a single patient. **(Right)** Noninvasive ECGI epicardial maps of LV and RV activation recorded from the same patient during **(A)** intrinsic rhythm (LBBB), **(B)** selective His bundle pacing, and **(C)** biventricular pacing. The color scale **(left)** shows that the late **(blue)** activation that occurs on the lateral wall of the LV during intrinsic activation is not seen with His bundle pacing: the LBBB pattern of activation is no longer present during His bundle pacing; a normal physiological ventricular pattern is seen instead. The small regions of **red**, early activation on the RV during his bundle pacing, may represent subtle nonselectivity of capture or interpolation of signal noise misidentified as activation. During biventricular pacing, activation spreads from early activation sites in both the RV and LV. ECG = electrocardiogram; ECGI = electrocardiographic imaging; LAD = left anterior descending artery; LBBB = left bundle branch block; LV = left ventricle/ventricular; RV = right ventricle/ventricular.

Both HBP and BVP significantly reduced the LVDI (−17.0 ms; 95% CI: −21.9 to −12.0 ms; p < 0.001; −5.73 ms; 95% CI: −11.1 to −0.3 ms; p =0.04). HBP reduced LVDI significantly more than BVP: the mean within-patient change in LVDI from BVP to HBP was −11.3 ms (95% CI: −16.8 to −5.6 ms; p < 0.001) (Figure 4). An example of epicardial activation maps and ECGs are shown in Figure 5.

### Hemodynamic responses

**Figure 6:**
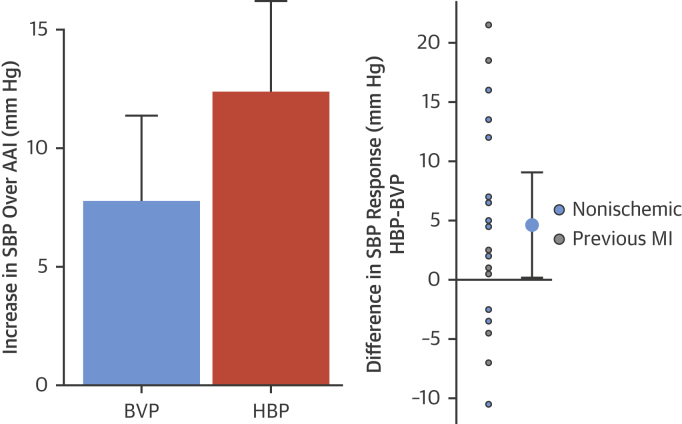
Hemodynamic Responses Acute improvement in systolic blood pressure is observed with both BVP and HBP **(left)**. His bundle pacing delivered significantly greater within-patient improvements in acute systolic blood pressure **(right)**. The 95% confidence intervals are displayed. Abbreviations as in Figures 1 and 2.

Both His bundle and BVP significantly increased acute SBP compared with AAI pacing (12.4 mm Hg; 95% CI: 8.6 to 16.2 mm Hg; p < 0.001; 7.8 mm Hg; 95% CI: 4.2 to 11.4 mm Hg; p < 0.001, respectively) in the 18 eligible patients in whom the full hemodynamic dataset was acquired. The improvement in SBP was significantly higher with HBP than with BVP (4.6 mm Hg; 95% CI: 0.2 to 9.1 mm Hg; p = 0.04) (Figure 6).

### Relationship between electrical and hemodynamic response

**Figure 7:**
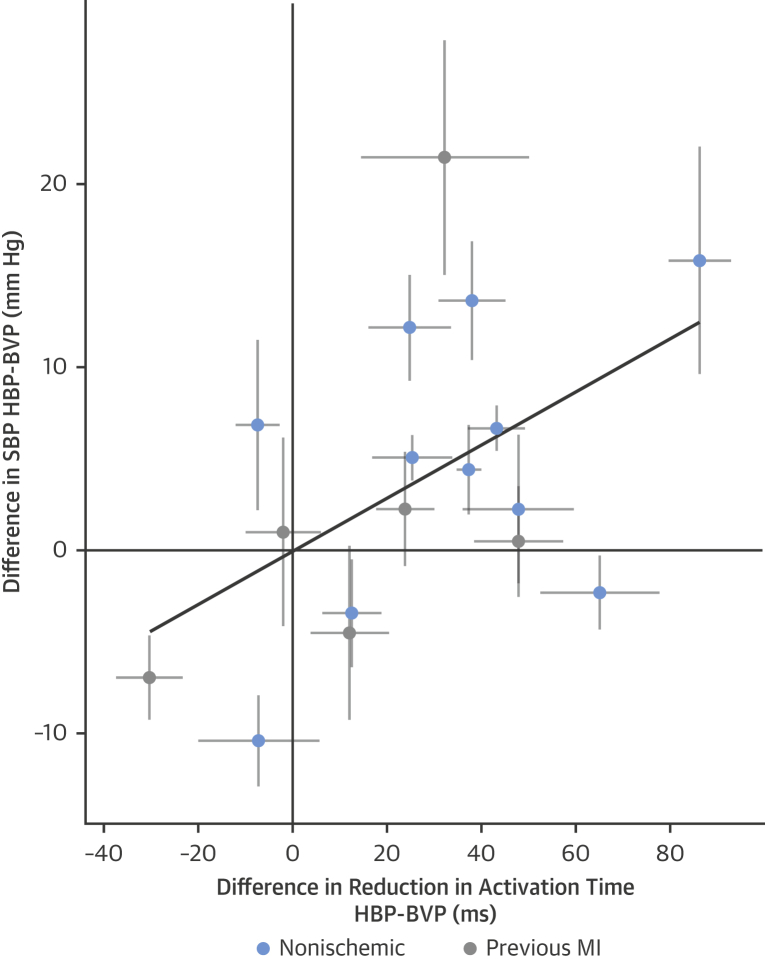
Correlation Between Electrical and Hemodynamic Responses Within-patient comparison of the difference in left ventricular activation time with HBP compared with BVP and the difference in acute SBP. **Thin black lines** = 95% confidence intervals in both axes. **Thick black line** = the regression line. Abbreviations as in Figures 1 and 2.

There was a significant correlation (R^2^ = 0.49, R = 0.70; p = 0.04) (Figure 7) between the individual patient incremental shortening of LVAT (LVAT-95) achieved by HBP over BVP against the incremental increase in blood pressure achieved by HBP over BVP. By contrast, the correlation between 12-lead QRSd reduction and hemodynamic response was not significant (p = 0.119).

### Response in patients without >10 ms LVAT-95 shortening from intrinsic

In the 4 patients where HBP did not reduce LVAT-95 by >10 ms, there was still an increase in SBP with HBP (4 mm Hg; 95% CI: −4.1 to 12.1 mm Hg; p = 0.20).

## Discussion

In this study we have, for the first time, quantified the acute effect of cardiac resynchronization therapy delivered with HBP, using high-precision hemodynamic measurements, and performed within-patient comparisons with BVP. We found that His resynchronization therapy delivers significantly greater improvements in acute hemodynamic response than BVP (Central Illustration). We used electroanatomic mapping to explore the mechanisms of this benefit. The additional improvements in hemodynamic function appear to be driven by improvements in ventricular activation time occurring as a result of more effective ventricular resynchronization.

**Central Illustration:**
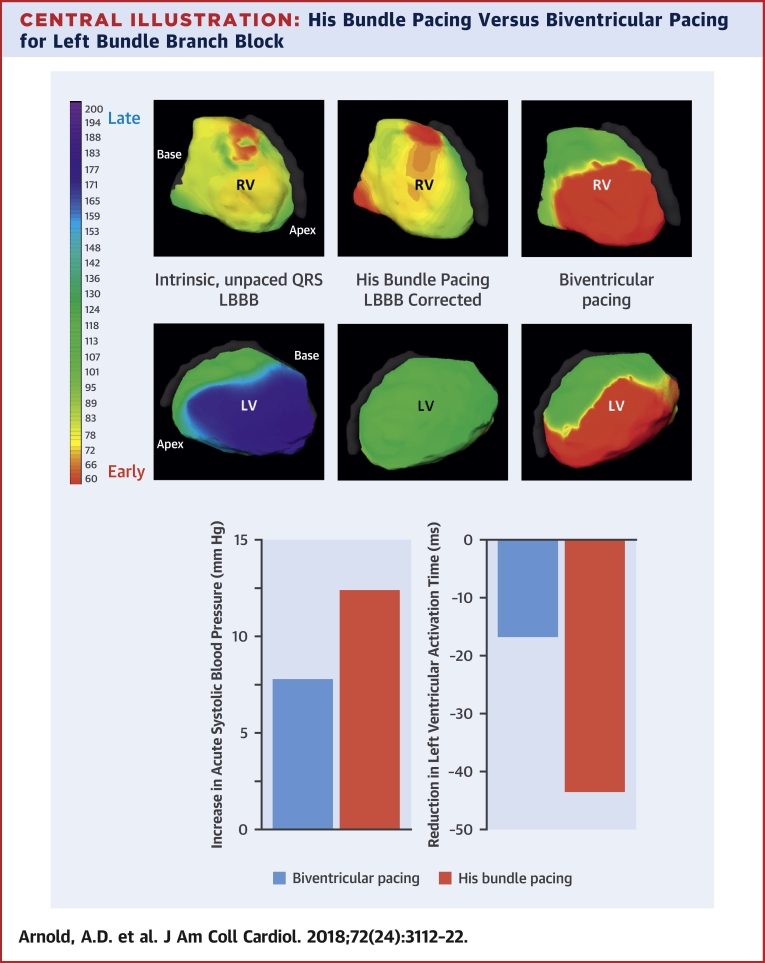
His Bundle Pacing Versus Biventricular Pacing for Left Bundle Branch Block **(Top)** Noninvasive epicardial RV and LV activation maps of intrinsic LBBB, His bundle pacing (HBP), and biventricular pacing (BVP). HBP produces a more physiological activation pattern than BVP. **(Bottom)** HBP produces a greater increase in acute systolic blood pressure and greater reduction in LV activation time than BVP. LBBB = left bundle branch block; LV = left ventricular; RV = right ventricular.

### Acute hemodynamic response

We found that HBP successfully delivered ventricular resynchronization in 83% of patients. In these patients, where HBP convincingly shortens LVAT, His resynchronization therapy resulted in significantly greater improvements in acute hemodynamic function compared with BVP. His resynchronization therapy delivered approximately a 60% increase in acute SBP, compared with BVP (4.6 mm Hg additional His-resynchronization SBP improvement over the 7.8 mm Hg with BVP).

BVP is the current gold standard method for delivering CRT. Hemodynamic studies have consistently demonstrated acute improvements when BVP is delivered to patients with LBBB and LV impairment 3, 24 and subsequent longer-term studies have confirmed reductions in morbidity and mortality. In LBBB, all-cause mortality is reduced by 34% (hazard ratio: 0.66; 95% CI: 0.55 to 0.78, from a 3,872-patient analysis) (2). If the 60% larger increase in SBP observed with His resynchronization therapy translates into longer-term endpoints, clinically important improvements in outcomes may ensue. Ideally, the increase in SBP would translate to an additional 20% reduction in mortality over BVP (60% of the 34% mortality reduction with BVP), which is similar to the mortality reduction observed with prognostic heart failure pharmacotherapy (25). This hypothesis needs to be tested in adequately powered randomized controlled trials, but the findings from this study provide justification and sample-size estimation for planning these studies.

### Mechanism of improvement with His resynchronization therapy

The correlation between activation time shortening and acute hemodynamic response suggests that the improved hemodynamic response with HBP is driven by more effective ventricular resynchronization (Figure 7).

His resynchronization therapy provided more than twice as much reduction in LVAT-95 and LVDI compared with BVP. BVP therapy appears to deliver some of its beneficial effect by improving atrioventricular timing (5), and the same would be expected of HBP, which may explain the smaller percentage improvement in hemodynamic response compared with the magnitude of intraventricular electrical synchronization.

The ventricular activation maps obtained during His pacing typically showed resolution of the LBBB activation pattern seen during intrinsic conduction. Figure 5 shows an example of LBBB resolution with selective HBP. This supports the concept that His resynchronization is achieved by recruiting LV conduction fibers, as suggested by Lustgarten et al. (9). By contrast, BVP produces nonphysiological activation patterns. Biventricular pacing is not a perfect resynchronization tool: relying on slow cell-to-cell conduction limits the degree of ventricular resynchronization that can be achieved. Indeed, BVP induces ventricular dyssynchrony when applied to patients with a narrow QRS (7), resulting in worse clinical outcomes (26).

Our finding that the degree of shortening of ventricular activation time is proportional to the improvement in function is potentially important. Ventricular activation could feasibly be quantified at the time of lead implantation. The His lead could then be positioned at the site of greatest activation time reduction, to facilitate maximal improvements in myocardial performance.

His pacing has other potential advantages compared with BVP. It does not require the use of contrast and is not limited by diaphragmatic capture or the constraints of the coronary sinus anatomy.

### Possible mechanisms by which HBP improves activation times

There are several possible explanations for how His Bundle pacing reduces activation time in bundle branch block. The simplest explanation is that the pacing lead is positioned distal to the site of block, thus recruiting distal conduction fibers that are longitudinally dissociated (electrical bypass) 27, 28. This would not explain how shortening is achieved when the lead is apparently located proximal to the block, because HBP is usually achieved from the right atrium, whereas the anatomic site of block may be below the bundle of His, within the ventricles. Therefore, alternative explanations argue that although the lead is not anatomically distal to the block, electrical energy is nevertheless applied to distal conduction fibers either through a large volume of myocardium encompassed by the pacing stimulus (virtual electrode hypothesis) or high energy (source–sink hypothesis). Although more local myocardium might be captured, increasing the size of the pseudo-delta wave, the majority of myocardium is activated by the restored His-Purkinje system. Another possible explanation is that distal fibers may unfurl back proximally, closer to the lead, resulting in retrograde activation up these fibers.

### Relationship with existing evidence

Our study expands upon the existing published reports regarding correction of LBBB by HBP with important new consequences. Lustgarten et al. (9) have performed a crossover comparison of permanent HBP and BVP, showing feasibility of permanent HBP in this population. They observed that, with respect to their effects on symptomatic, functional, and echocardiographic outcomes, HBP was not inferior to BVP (9). They also displayed an example of convincing acute hemodynamic improvement with HBP. Our study extends this pioneering work, by applying high precision measurements systematically. We found that HBP delivered a significantly greater improvement in acute hemodynamic function than BVP. Lustgarten et al. (9) observed a narrower QRS with selective, but not nonselective, HBP compared with BVP. We used high-resolution ECGI measurements, allowing us to assess LVAT, which is less likely to be influenced by nonselective capture. We found a close correlation between LV activation time and acute hemodynamic response. Further studies have demonstrated that permanent His resynchronization therapy is technically possible in patients with LBBB and heart failure with very low and stable thresholds 8, 10, 29. Therefore, it is now feasible to perform clinical trials to test whether the acute improvements in ventricular resynchronization and hemodynamic function we observed with His resynchronization therapy, translate into longer-term benefits.

### Study limitations

The study has many fewer patients than a long-term event trial. This is because when endpoints are continuous variables, rather than event counting, each patient contributes far more power. By using high-precision hemodynamic assessment and high-resolution electrical mapping, we were able to confidently assess the individual patient responses and correlate the electrical response to the hemodynamic response. The source of precision and statistical power is the large numbers of measurements per patient, which provided narrow error-bar measurements. This study was not specifically powered to discriminate different responses of ischemic and nonischemic cardiomyopathies to HBP. However, it was encouraging that we observed successful His resynchronization in both.

This was an acute hemodynamic study. We do not know if the significant improvements in activation time and acute hemodynamic response we observed will translate into better hemodynamic outcomes with longer-term follow-up. The previous experience with BVP supports the concept that short-term resynchronization induced improvements translate into longer-term benefits. However, this question can only be answered by randomized controlled trials with long-term follow up. The findings from this study provide justification for these trials. Ventricular remodeling in response to chronic electrical resynchronization may contribute to the long-term clinical improvements seen with BVP. It is plausible that superior ventricular resynchronization with HBP may lead to greater long-term effects on ventricular remodeling, but this needs to be tested in dedicated studies.

Biventricular pacing is reported to be variable in its response rate (30), so it possible that we could have recruited an unusually high proportion of “nonresponders.” However, the QRSd reductions achieved by BVP (−17 ms; 95% CI: −27.3 to 7.1 ms) in this study are greater than those seen in BVP studies (−6.6 ± 27.8 ms), even when those studies excluded nonresponders (−11.9 ± 25.1 ms) (6).

Although noninvasive ECGI mapping provides high-resolution data of activation and allows segregation of regional activation, there are potential problems including assumptions of static geometry and interpolation accuracy (31) (Online Appendix).

We did not observe successful ventricular resynchronization with HBP in all patients in this study, which is consistent with the findings of other studies (8). As this was an acute hemodynamic study of temporary HBP, we did not employ active fixation, which is used in permanent HBP. Active fixation may result in lower thresholds because the screw of the active lead acts as the cathode, and therefore, when it penetrates the His bundle, it may allow direct stimulation of the conduction fibers. However, some patients with QRS prolongation may not be amenable to ventricular resynchronization with His pacing, for example, patients where QRS prolongation occurs due to myocyte uncoupling.

## Conclusions

CRT delivered using HBP appears to be a very promising alternative to BVP in patients with LBBB and heart failure. It can deliver larger reductions in ventricular activation time, which leads to significantly greater improvements in acute hemodynamic function. The magnitude of these improvements suggest that His resynchronization therapy has the potential to produce better clinical outcomes than BVP.

Perspectives **COMPETENCY IN PATIENT CARE AND PROCEDURAL SKILLS:** In patients with heart failure and LBBB, His bundle pacing is associated with more effective ventricular resynchronization and greater improvement in systolic blood pressure than cardiac resynchronization therapy with biventricular pacing.

**TRANSLATIONAL OUTLOOK:** Randomized trials comparing His bundle pacing against biventricular pacing are needed to guide selection of optimum device-based therapy for patients with heart failure and LBBB.
