# Supplementary material for: His Resynchronization Versus Biventricular Pacing in Patients With Heart Failure and Left Bundle Branch Block
Source: J Am Coll Cardiol. 2018 Dec 18;72(24):3112–22. doi: 10.1016/j.jacc.2018.09.073 (PMC6290113; doi:10.1016/j.jacc.2018.09.073)
Supplement: Online Appendix [file mmc1.docx]

## **Supplemental Material**

## **High Precision Hemodynamic Protocol**

The hemodynamic assessment was performed as follows. Eight beats of blood-pressure data were acquired and averaged during atrial-only pacing (AAI: unpaced QRS complex) immediately before transitioning to His bundle or biventricular pacing. The rate was fixed throughout at approximately 5 beats per minute above the sinus rate. Immediately following the transition a further eight beats were acquired and averaged. The difference between mean systolic blood pressure before and after transition (the relative change from atrial pacing) is a hemodynamic response to the pacing intervention. AAI pacing was then restored and a further 8 beats were averaged to create a further transition and response to pacing. This was repeated a minimum of a further three times so that a total of at least 8 transitions between AAI pacing and biventricular or His bundle pacing were acquired (**Figure 1**). The mean relative change for each tested setting was calculated from a minimum of 8 transitions. If ventricular ectopy was observed, additional transitions were performed. All hemodynamic measurements are subject to variability, which occurs due to respiration, change in sympathetic tone as well as inevitable spontaneous variability inherent to any biological measurement, necessitating this approach. Analysis was automated and no data points were manually removed.

This protocol was performed for a full range of AV delays for each tested intervention (His bundle pacing and biventricular pacing), from 40ms in 40ms increments until intrinsic conduction broke through. This allowed calculation of the peak hemodynamic response as LV filling variation with changing AV delay has a large impact on hemodynamic response. The entire protocol was performed for both His bundle pacing and biventricular pacing as the AV delay associated with peak response may differ between these interventions. Varying VV delay typically has a small hemodynamic impact and so was not assessed (1).

For biventricular pacemaker implantation the right ventricular lead was positioned in the RV apex and a quadripolar coronary sinus (CS) lead was positioned in the most clinically suitable lateral ventricular branch, the right atrial lead was positioned in the right atrial appendage (RAA). The left ventricular lead pacing vector was selected on the basis of avoiding phrenic capture or high threshold in accordance with standard practice at our institution. Atrioventricular (AV) sequential pacing for biventricular pacing was performed via the device.

Using this protocol, we used change in acute systolic blood pressure to measure hemodynamic response. Systolic blood pressure has several features that make it well suited for assessing the acute impact of tested pacing interventions on cardiac function. Firstly, it is a down-stream extra-cardiac measurement and therefore assesses overall net effect pacing interventions have on overall cardiac function. Acute changes in systolic blood pressure closely reflect changes in aortic flow (2). Since changes in neurohormonal vasoregulation take at least several seconds to occur, the instantaneous change in resistance, R, is zero (R_2_ = R_1_). Instantaneous changes in pressure, (ΔP = P_2_ – P_1_), are thus proportional to instantaneous changes in cardiac output (ΔP = CO_2_*R_1_ – CO_1_*R_1_ = ΔCO*R_1_ ∴ ΔP α ΔCO)(2,3). Secondly, systolic blood pressure measurements are highly reproducible, with a high signal-to-noise ratio, if beat-by-beat systolic blood pressure is acquired and multiple measurements are made and compared to a reference setting (2,3). Thirdly, in patients with heart failure sustained increases in blood pressure are associated with improved outcomes (4) and in randomized trials when biventricular pacing has been applied to patients with LBBB and heart failure it results in sustained increases in systolic blood pressure (5).

**Power calculation**

This method generates accurate, reproducible peak hemodynamic response estimates with sufficiently narrow confidence intervals that clinically important, statistically significant, within-patient differences between interventions can be identified from a relatively small number of subjects. We determined that a 3 mmHg incremental improvement of systolic blood pressure with His bundle pacing over biventricular pacing would be likely to be clinically important. A 3 mmHg improvement represents 50% of the mean 6 mmHg improvement which has been observed in previous studies when biventricular pacing is delivered to patients with LBBB and heart failure (6,7). If this translates in to an additional 50% improvement in long term clinical outcomes, then the effect size would be on par with those with ACE inhibitors for patients with heart failure (8). This would represent a clinically meaningful improvement. We used a predicted standard deviation for this effect of 3.72 mmHg derived from the hemodynamic effect of biventricular pacing over atrial pacing using the same high-precision methodology we used in this study (6). With a power of 80% and significance level of 0.05, 15 subjects were required for the study to be adequately powered to determine a 3 mmHg improvement. Therefore 15 patients exhibiting His Resynchronization would need to be recruited. The correction rate of bundle branch block by His bundle pacing has been reported to be as high as 83% (9), however as we were performing temporary His bundle pacing we assumed a lower correction rate of 75% and therefore aimed to recruit 20 patients, with further patients to be recruited if the entire dataset was not recorded in those initially recruited.

**Non-Invasive Epicardial Mapping (ECGI): Methods And Limitations**

The ECGI methodology has previously been described and validated (10,11). Body-surface potential data are obtained by the 252-electrode vest and combined with radiologically derived anatomical data using the ECGI system which provides a solution to the inverse problem to reconstruct unipolar epicardial electrograms (EGMs). Using custom in-house software, we extracted the epicardial electrograms and their anatomical locations during beats of interest (AAI pacing with unpaced QRS, His bundle pacing, biventricular pacing). Activations from individual electrodes were temporally annotated based on the most negative dv/dt of the EGM and visualized on the three-dimensional cardiac model derived from the subject’s individual CT thorax. This allows calculation of activation times from the left and right ventricles individually.

There are limitations to this method. Anatomical data is acquired at a single time-point but electrical measurements are made over a period of time. Therefore, an assumption of static cardiac and body surface geometry is necessary. Furthermore, there is debate regarding the maximum achievable reconstruction resolution as well as the optimal number and positioning of body-surface electrodes. However, this is most problematic when ECGI is used to identify locations for ablation rather than measurements of activation time and all such issues are likely to affect atrial, biventricular and his bundle pacing measurements to similar extents.

**References for Supplementary Material**

1. Sohaib SA, Kyriacou A, Jones S et al. Evidence that conflict regarding size of haemodynamic response to interventricular delay optimization of cardiac resynchronization therapy may arise from differences in how atrioventricular delay is kept constant. EP Europace 2015;17:1823-1833.

2. Manisty CH, Al-Hussaini A, Unsworth B et al. The acute effects of changes to AV delay on blood pressure and stroke volume: potential implications for design of pacemaker optimization protocols. Circulation: Arrhythmia and Electrophysiology 2011:CIRCEP. 111.964205.

3. Whinnett Z, Davies J, Willson K et al. Haemodynamic effects of changes in atrioventricular and interventricular delay in cardiac resynchronisation therapy show a consistent pattern: analysis of shape, magnitude and relative importance of atrioventricular and interventricular delay. Heart 2006;92:1628-1634.

4. Raphael CE, Whinnett ZI, Davies J et al. Quantifying the paradoxical effect of higher systolic blood pressure on mortality in chronic heart failure. Heart 2009;95:56-62.

5. Cleland JG, Daubert J-C, Erdmann E et al. The effect of cardiac resynchronization on morbidity and mortality in heart failure. New England Journal of Medicine 2005;352:1539-1549.

6. Sohaib SA, Wright I, Lim E et al. Atrioventricular optimized direct His bundle pacing improves acute hemodynamic function in patients with heart failure and PR interval prolongation without left bundle branch block. JACC: Clinical Electrophysiology 2015;1:582-591.

7. Whinnett ZI, Sohaib SA, Mason M et al. Multicenter randomized controlled crossover trial comparing hemodynamic optimization against echocardiographic optimization of AV and VV delay of cardiac resynchronization therapy: the BRAVO trial. JACC: Cardiovascular Imaging 2018:2575.

8. Investigators* S. Effect of enalapril on survival in patients with reduced left ventricular ejection fractions and congestive heart failure. New England Journal of Medicine 1991;325:293-302.

9. Teng AE, Lustgarten DL, Vijayaraman P et al. Usefulness of His bundle pacing to achieve electrical resynchronization in patients with complete left bundle branch block and the relation between native QRS axis, duration, and normalization. American Journal of Cardiology 2016;118:527-534.

10. Ramanathan C, Ghanem RN, Jia P, Ryu K, Rudy Y. Noninvasive electrocardiographic imaging for cardiac electrophysiology and arrhythmia. Nature medicine 2004;10:422.

11. Rudy Y. Noninvasive electrocardiographic imaging of arrhythmogenic substrates in humans. Circulation research 2013;112:863-874.
